# Draft Genome Sequences of Eight Isolates of Beauveria bassiana of Neotropical Origin

**DOI:** 10.1128/mra.00267-22

**Published:** 2022-06-06

**Authors:** Ruth Castro-Vásquez, Stefany Solano-González, Ramón Molina-Bravo, Mauricio Montero-Astúa

**Affiliations:** a Centro de Investigación en Biología Celular y Molecular, Universidad de Costa Rica, San José, Costa Rica; b Universidad Nacional, Escuela de Ciencias Biológicas, Laboratorio de Bioinformática Aplicada (LABAP), Heredia, Costa Rica; c Universidad Nacional, Escuela de Ciencias Agrarias, Programa de Biotecnología Vegetal y Recursos Genéticos para el Fitomejoramiento (BIOVERFI), Heredia, Costa Rica; d Escuela de Agronomía, Universidad de Costa Rica, San José, Costa Rica; Vanderbilt University

## Abstract

Beauveria bassiana, a well-known entomopathogenic fungus, has a worldwide distribution; however, genomes of isolates from the Neotropical region are scarce. Here, we report the draft genome sequences of eight *B.* bassiana isolates from Costa Rica, Puerto Rico, and Honduras.

## ANNOUNCEMENT

The fungus Beauveria bassiana is a well-known biocontrol agent with a global distribution and a wide range of hosts (1). Several B. bassiana genomes have been reported, mostly from Asia (2–7). Here, we report the genome assemblies of eight isolates from Central America and the Caribbean. B. bassiana has an important role in agricultural ecosystems in the tropics (8), but more studies are needed to understand its ecological and genetic diversity (9).

We report five fungal isolates from Costa Rica, two from Puerto Rico, and one from Honduras, available at the Entomopathogenic Fungi Collection (EFC) at the National University of Costa Rica (UNA). A previous characterization suggested high diversity within the eight isolates (10).

A standard chloroform-isoamyl alcohol DNA extraction was performed using 100 mg of 10- to 15-day-old monosporic mycelia grown on potato dextrose agar (PDA) (11). Libraries were prepared using a WaferGen robotic DNA library prep. Whole-genome sequencing was performed at the Center for Genomic Research and Biocomputing at Oregon State University using a 2 × 150-bp paired-end HiSeq 3000 Illumina platform (Illumina, Inc., San Diego, CA), according to the manufacturer’s protocol at 50× coverage.

Data quality and adapter removal were done with FastQC and Trimmomatic v0.38 (12), respectively. Arguments used for Trimmomatic: *TruSeq2-PE.fa* as adapters file, *leading* and *trailing* were set as 3, *sliding window* was set at 4:15, and *minlen* was set at 36.

*De novo* assembly was performed with SPAdes v3.13.1 (13, 14), and genome assemblies were compared and evaluated using Quast v5.0.2 (15) against the Beauveria bassiana ARSEF 2860 (GenBank accession no. ADAH00000000.1). BUSCO v3.0.1 (16) was implemented to check for genome completeness against eukaryota_odb9, using Fusarium as the species argument. BUSCO results were relatively homogenous throughout the genomes.

For draft optimization of the assembled genomes, Scaffolder MeDuSa v1.6 (17) was used against B. bassiana ARSEF 8028 (GenBank assembly no. GCA_001682635.1) and Bv062 (GenBank assembly no. GCA_003337105.1). Gene calling for these improved assemblies was done using Augustus v2.6.1 against an Aspergillus oryzae training set. The genome assemblies provided in this study ranged from 32.1 to 34.8 Mb, genome completeness was estimated to be 82.23 to 86.94%, and predicted coding proteins ranged between 9,500 and 10,453 (Table 1). These assemblies and their annotations will aid insecticidal mechanism exploitations tailored toward Neotropical pest management and biotech applications.

**TABLE 1:** Summary statistics for the genome assembly of eight B. bassiana isolates of Neotropical origin

Isolate	Genome size (Mb)	Total no. of reads	No. of contigs	*N*_50_ Mb	% G +C content	% of BUSCO conserved gene set for eukaryote Fusarium graminearum	No of predicted proteins	GenBank accession no.
BV-ECA0	32.8	24,821,590	420	2.07	50.70	97	9,593	JACVNG000000000
BV-ECA1	33	31,240,515	877	2.94	50.71	97	9,636	JACVNF000000000
BV-ECA13	32.8	22,937,002	202	3.90	50.69	96	9,566	JACVNE000000000
BV-ECA26	32.16	39,078,169	76	1.10	50.79	97	9,465	JACVND000000000
BV-ECA27	32.97	41,021,666	614	1.49	50.71	97	9,648	JACVNC000000000
BV-ECA31	33	27,655,085	69	1.43	50.08	97	9,546	JACVNB000000000
BV-ECA43	34.5	27,606,205	551	0.89	50.62	97	10,210	JACVNA000000000
BV-ECA44	34.8	26,336,978	1,118	0.95	50.61	97	10,453	JACVMZ000000000
ARSEF 2860	33.6	NA^*a*^	1,229	0.73	51.50	NA	10,364	ADAH00000000.1
ARSEF 8028	33.6	NA	1,229	0.73	51.50	NA	10,210	GCA_001682635.1

a NA, not applicable.

### Data availability.

This whole-genome shotgun project has been deposited at DDBJ/ENA/GenBank (Table 1). The versions described correspond to the first version. Illumina sequence data were deposited under accession no. SRR12774113 to SRR12774120 (BioProject no. PRJNA658593).
